# Supplementary material for: Evaluating generative AI integration in Saudi Arabian education: a mixed-methods study
Source: PeerJ Comput Sci. 2024 Feb 16;10:e1879. doi: 10.7717/peerj-cs.1879 (PMC10909195; doi:10.7717/peerj-cs.1879)
Supplement: Supplemental Information 2 — Survey used in research data collection [file peerj-cs-10-1879-s002.docx]

## Survey Questionnaire

Thank you for contributing in our survey. Please answer to all of the questions. Your responses will be used in an academic study only.

1. **Gender**

 Male

 Female

1. **Age**

 18-25

 26-34

 35-44

 More than 45

1. **Do you know about Generative AI such as ChatGPT?**

 Yes

 NO

1. **Do you use Generative AI such as ChatGPT in your teaching courses?**

 Yes

 NO

**Educators’ Perceptions of GAI Integration in Education**

Please rate your level of agreement to the following questions

| **Question** | **Strongly Disagree** | **Disagree** | **Neutral** | **Agree** | **Strongly Agree** |
| --- | --- | --- | --- | --- | --- |
| 1. Increases academic achievement (e.g., grades). | ☐ | ☐ | ☐ | ☐ | ☐ |
| 2. Results in students neglecting important resources. | ☐ | ☐ | ☐ | ☐ | ☐ |
| 3. Is effective because I believe I can implement it. | ☐ | ☐ | ☐ | ☐ | ☐ |
| 4. Promotes student collaboration. | ☐ | ☐ | ☐ | ☐ | ☐ |
| 5. Promotes the development of communication skills. | ☐ | ☐ | ☐ | ☐ | ☐ |
| 6. Is a valuable instructional tool. | ☐ | ☐ | ☐ | ☐ | ☐ |
| 7. Makes teachers feel more competent as educators. | ☐ | ☐ | ☐ | ☐ | ☐ |
| 8. Is an effective tool for students of all abilities. | ☐ | ☐ | ☐ | ☐ | ☐ |
| 9. Enhances my professional development. | ☐ | ☐ | ☐ | ☐ | ☐ |
| 10. Eases the pressure on me as a teacher. | ☐ | ☐ | ☐ | ☐ | ☐ |
| 11. Motivates students to get more involved in learning. | ☐ | ☐ | ☐ | ☐ | ☐ |
| 12. Should reduce the number of teachers employed. | ☐ | ☐ | ☐ | ☐ | ☐ |
| 13. Will increase the amount of stress and anxiety. | ☐ | ☐ | ☐ | ☐ | ☐ |
| 14. Requires extra time to plan learning activities. | ☐ | ☐ | ☐ | ☐ | ☐ |
| 15. Improves student learning of critical concepts. | ☐ | ☐ | ☐ | ☐ | ☐ |
